# A Novel Homozygous In-Frame Deletion in Complement Factor 3 Underlies Early-Onset Autosomal Recessive Atypical Hemolytic Uremic Syndrome - Case Report

**DOI:** 10.3389/fimmu.2021.608604

**Published:** 2021-06-24

**Authors:** Shirley Pollack, Israel Eisenstein, Adi Mory, Tamar Paperna, Ayala Ofir, Hagit Baris-Feldman, Karin Weiss, Nóra Veszeli, Dorottya Csuka, Revital Shemer, Fabian Glaser, Zoltán Prohászka, Daniella Magen

**Affiliations:** ^1^ Pediatric Nephrology Institute, Ruth Children’s Hospital, Haifa, Israel; ^2^ Rappaport Faculty of Medicine, Technion - Israel Institute of Technology, Haifa, Israel; ^3^ Genetic Institute, Haifa, Israel; ^4^ Research Laboratory, Department of Internal Medicine and Haematology, and MTA-SE Research Group of Immunology and Hematology, Hungarian Academy of Sciences and Semmelweis University, Budapest, Hungary; ^5^ Laboratory of Molecular Medicine, Rappaport Faculty of Medicine, Technion - Israel Institute of Technology, Haifa, Israel; ^6^ Bioinformatics Knowledge Unit, The Lorry I. Lokey Interdisciplinary Center for Life Sciences and Engineering, Technion-Israel Institute of Technology, Haifa, Israel

**Keywords:** atypical hemolytic uremic syndrome (aHUS), complement factor C3, eculizumab, alternative complement pathway, homozygous deletion

## Abstract

**Background and Objectives:**

Atypical hemolytic uremic syndrome (aHUS) is mostly attributed to dysregulation of the alternative complement pathway (ACP) secondary to disease-causing variants in complement components or regulatory proteins. Hereditary aHUS due to C3 disruption is rare, usually caused by heterozygous activating mutations in the *C3* gene, and transmitted as autosomal dominant traits. We studied the molecular basis of early-onset aHUS, associated with an unusual finding of a novel homozygous activating deletion in C3.

**Design, Setting, Participants, & Measurements:**

A male neonate with eculizumab-responsive fulminant aHUS and C3 hypocomplementemia, and six of his healthy close relatives were investigated. Genetic analysis on genomic DNA was performed by exome sequencing of the patient, followed by targeted Sanger sequencing for variant detection in his close relatives. Complement components analysis using specific immunoassays was performed on frozen plasma samples from the patient and mother.

**Results:**

Exome sequencing revealed a novel homozygous variant in exon 26 of *C3* (c.3322_3333del, p.Ile1108_Lys1111del), within the highly conserved thioester-containing domain (TED), fully segregating with the familial disease phenotype, as compatible with autosomal recessive inheritance. Complement profiling of the patient showed decreased C3 and FB levels, with elevated levels of the terminal membrane attack complex, while his healthy heterozygous mother showed intermediate levels of C3 consumption.

**Conclusions:**

Our findings represent the first description of aHUS secondary to a novel homozygous deletion in C3 with ensuing unbalanced C3 over-activation, highlighting a critical role for the disrupted C3-TED domain in the disease mechanism.

## Introduction

Atypical hemolytic uremic syndrome (aHUS) is a heterogeneous group of acquired and hereditary thrombotic microangiopathy (TMA), manifested by microangiopathic hemolytic anemia, thrombocytopenia and acute kidney injury, which are usually not preceded by the diarrheal prodromal phase of Shiga Toxin-related HUS. Most forms of aHUS are associated with dysregulation of the alternative complement pathway (ACP), resulting in complement-mediated endothelial cell injury, with ensuing end-organ tissue damage (1, 2).

To date, about two thirds of patients with aHUS carry identifiable mutations or likely-pathogenic risk variants in genes encoding complement pathway proteins (2–5). While loss-of-function mutations are commonly implicated in genes encoding regulatory complement components, including complement Factor H (*CFH*), complement Factor I (*CFI*) and membrane cofactor protein (MCP, *CD46*), gain-of-function mutations are usually associated with complement Factor B (*CFB*) and *C3* (6–8). In conjunction with genetic predisposition, acquired autoantibodies against Factor H (FH) have been implicated in the pathogenesis of aHUS in approximately 10% of cases, and are mostly attributed to genomic rearrangements or deletions in *CFH*/*CFHR1/CFHR3/CFHR4* genes within the regulators of complement activation gene cluster (9, 10). In addition to proteins directly related to the complement cascade, increasing evidence highlights the role of coagulation factors in the pathogenesis of aHUS, including thrombomodulin (*THBD*), which regulates Factor I-mediated C3b inactivation, and plasminogen (*PLG*), which enhances plasmin-mediated fibrinolysis and thrombi degradation (11, 12). Finally, mutations in *DGKE*, encoding diacylglycerol kinase-ϵ (DGKE) have been linked with infantile-onset aHUS, accompanied by nephrotic syndrome and chronic kidney disease (CKD), through an as yet unresolved mechanism (8, 13).

Given the central role of ACP over-activation in the pathogenesis of aHUS, a favorable response to targeted blockade of the complement cascade is highly conceivable. Eculizumab is a monoclonal antibody directed against the human C5 complement component, causing inhibition of C5a release, thereby preventing downstream generation of the membrane attack complex (MAC), C5b-9. As such, it is indicated in acute episodes of aHUS, and as prophylaxis in susceptible patients (3, 14).

In this report we describe a male neonate with a life-threatening presentation of aHUS, associated with a novel homozygous in-frame deletion in the *C3* gene. Complement profile analysis suggests unbalanced activation of the ACP, which is further supported by a favorable response to eculizumab therapy. To the best of our knowledge, this is the first report of a homozygous deletion in *C3* associated with severe neonatal presentation of aHUS, secondary to C3 over-activation.

## Clinical Presentation and Workup

The patient is the 5^th^ child of healthy, 1^st^-degree cousins of Arab-Muslim origin, born at term after normal pregnancy and delivery. Detailed family history was negative for renal, hematological, autoimmune or recurrent infectious disorders. He was admitted to a local hospital at the age of 3 days for evaluation of extensive jaundice, anemia, thrombocytopenia, and impaired kidney function. Following fulminant renal failure and acute respiratory insufficiency, he was urgently transferred to our care for further evaluation and management. On admission, the infant displayed clinical signs of encephalopathy, accompanied by fluid overload and pulmonary congestion secondary to anuric renal failure, which necessitated mechanical ventilation and renal replacement therapy.

Initial laboratory investigation was compatible with TMA, as evidenced by normocytic anemia, severe thrombocytopenia, significant schistocytosis on peripheral blood smear, a negative Coombs test, and extremely elevated lactate dehydrogenase (LDH) levels. A thorough microbiological workup was negative for an identifiable infectious trigger. Plasma ADAMTS-13 activity was normal, and anti-ADAMTS-13 antibodies were undetected, thereby excluding thrombotic thrombocytopenic purpura (TTP). Coagulation tests excluded diffuse intravascular coagulation (DIC). Metabolic screen showed normal blood levels of homocysteine, methionine and cobalamin C, with no evidence of organic aciduria, thereby excluding cobalamin C deficiency. Serum C3 levels were repeatedly extremely low, with normal C4 levels. A connective tissue disorder panel (collagenogram) was negative. Imaging studies, including renal echo-Doppler, brain ultrasonography and echocardiography were unremarkable.

With a working diagnosis of aHUS, the patient was managed with eculizumab therapy, in conjunction with mechanical ventilation, daily hemodialysis, red blood cell transfusions and prophylactic penicillin therapy. Within several days of eculizumb initiation, the infant showed dramatic clinical and laboratory improvement, followed by rapid neurological, hematological, respiratory and renal recovery. Currently, at the age of 2 years, the child is in sustained remission from aHUS under prophylactic eculizumab therapy, with a history of two reversible aHUS relapses following non-adherence to prescribed eculizumab prophylaxis. Each relapse appeared after 10-14 days delay in scheduled eculizumab therapy, manifesting with thrombotic microangiopathy and acute renal failure. Sequelae of his fulminant presenting episode of aHUS include arterial hypertension, non-nephrotic range proteinuria with normal glomerular filtration rate (GFR), and gross motor developmental delay. His serum C3 levels are constantly low. He does not exhibit increased susceptibility to pyogenic bacterial infections, as reported in patients with homozygous loss-of-function variants in *C3* which cause C3 deficiency (15, 16), although infections may have been prevented by prophylactic antibiotic treatment.

## Materials and Methods

### Genetic Analysis

Genomic DNA was extracted from the patient’s peripheral lymphocytes for clinical exome sequencing, using the TruSight One Sequencing panel (Illumina, Inc., San Diego, CA, USA), according to manufacturer’s instructions. Direct Sanger sequencing was performed on the coding regions and intron-exon boundaries of *CFH* (exons 1-9 & 11-23), *CFI* (exons 1-13), *MCP* (CD46, exons 1-14), *C3* (exons 1-41), and *CFB* (exons 1-18); *THBD* (exon 1).

Multiplex Ligation-Dependent Probe Amplification (MLPA) analysis was performed using a commercially available assay (P236-A3 ARMD probemix, MRC-Holland).

All available family members were Sanger sequenced for the *C3* variant and for additional variations and risk polymorphisms identified in the patient.

Pathogenicity of identified sequence variants was classified according to joint consensus recommendations by the American College of Medical Genetics and Genomics and the Association for Molecular Pathology (17).

### Complement Profile Analysis

Levels of complement components, regulators and FH autoantibodies were determined on frozen plasma samples of the patient and his mother, according to previously published methods (18). Fresh plasma samples for complement analysis on additional family members were unavaliable.

### C3b-FH Complex Modelling

Structural analysis of C3b-FH complex was based on the PDB 3OXU complex structure (19). Interfacial amino acids of C3b were defined as those within a distance < 5 Å of any atom of Factor H. The energy of the C3b-FH complex was computed with pyDock bindEy module for protein structure prediction (20), both for the wild-type and the inferred mutant C3 protein structure. Molecular graphics and analyses were performed with the UCSF ChimeraX program, developed by the Resource for Biocomputing, Visualization, and Informatics at the University of California, SF, with support from National Institutes of Health R01-GM129325 and the Office of Cyber Infrastructure and Computational Biology, National Institute of Allergy and Infectious Diseases (21).

## Results

### Genetic Analysis

Sequence analysis of the patient revealed a homozygous in-frame deletion of 12bp in exon 26 of the *C3* gene (c.3322_3333del, p.Ile1108_Lys1111del) resulting in deletion of highly conserved four amino acids (ILEK) from the inferred protein sequence. The four deleted residues are predicted to be located in the α-chain of the encoded C3 protein, within its highly conserved thioester-containing domain (TED) (NCBI conserved domains). The p.Ile1108_Lys1111del-C3 variant was absent from public variation data bases [dbSNP; 1000 Genomes Projects; gnomAD; The Greater Middle East Variom Project (GME)], and was predicted harmful by mutation prediction software (Mutation Taster).

**Figure 1:**
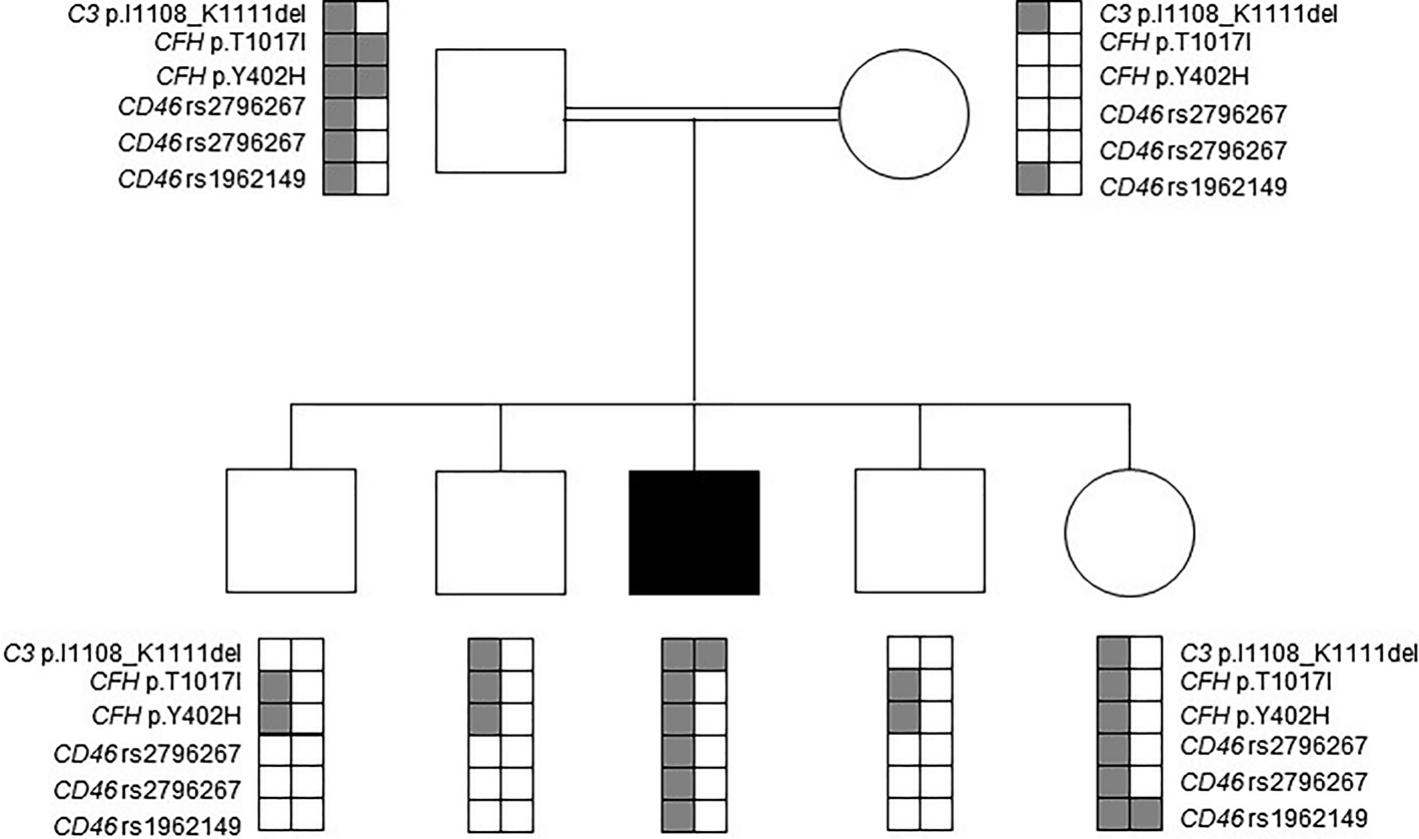
Mutation analysis in the patient’s nuclear family. Affected individual is marked by a black filled square. Rare alleles of the rs2796267, rs2796268 and rs1962149 polymorphisms and their haplotype referred as *MCP*ggaac were reported to be a risk factor for developing aHUS.

Several additional common polymorphisms and variants of unknown significance were identified in complement encoding genes of the patient (**Figure 1**). In detail, he was found to be heterozygous for a substitution in the *CFH* gene (c.3050C>T, p.Thr1017Ile). This variant has been previously classified as likely benign, based on comparable allele frequencies between healthy individuals and aHUS patients, combined with ambiguous prediction of pathogenicity, according to *in-silico* prediction software analysis (22). Further identified heterozygous polymorphisms in the patient included the *CFH* Y402H risk factor for dense deposit disease, as well as the *CD46* rs2796267, rs2796268 and rs1962149 polymorphisms, referred as part of the *MCP*ggaac risk haplotype for aHUS (**Figure 1**).

MLPA analysis excluded deletions or duplications within the Regulators of Complement Activation (RCA) gene cluster (*CFH, CFHR1, CFHR2, CFHR3* and *CFHR5*) on chromosome 1q31.3, as underlying the disease.

Targeted sequencing of all healthy close relatives excluded homozygosity for the p.Ile1108_Lys1111del-C3 variant. Heterozygosity for the *MCP*ggaac risk haplotype was identified in the healthy father and sister. The rare allele of the *CFH* Y402H risk polymorphism was identified in the healthy father, sister and brothers, but not in the healthy mother (**Figure 1**).

### Complement Profile Analysis

As shown in **Table 1**, complement analysis of the patient indicated critically low C3 level, deficient activity of all three complement pathways, decreased complement factor B (FB) level, and increased level of the terminal pathway activation marker. C1q, C4, FI and FH levels were within or slightly below the reference range. Anti-FH antibody titer was negative, below the cut-off.

**Table 1:** Complement profile of the patient and his mother.

	Patient	Healthy mother	Reference range
**ADAMTS13 activity**	99%	94%	67-150%
**Total complement activity,** **classical pathway (hemolytic test)**	0 CH50/ml	37 CH50/ml	48-103 CH50/ml
**Total complement activity, alternative pathway (WIELISA-Alt)**	0%	59%	70-125%
**Total complement activity,** **lectin pathway (WIELISA-LP):**	0%	4%	70-125%
**C3**	0.15 g/L	0.57 g/L	0.9-1.8 g/L
**C4**	0.21 g/L	0.28 g/L	0.15-0.55 g/L
**Factor H antigen**	222 mg/L	309 mg/L	250-880 mg/L
**Factor I antigen**	73%	87%	70-130%
**Factor B antigen**	39%	93%	70-130%
**Anti- factor H IgG autoantibody**	107 AU/mL	35 AU/mL	<110 AU/mL
**C1q antigen**	54 mg/mL	107 mg/mL	60-180 mg/mL
**Anti-C1q IgG autoantibody**	9 U/mL	3 U/mL	<52 U/mL
**sC5b-9 (terminal complement complex)**	456 ng/mL	239 ng/mL	110-252 ng/mL

Taken together, complement profile analysis of the patient suggests spontaneous activation of the p.lle1108_Lys111de-C3 protein by FB, resulting in ongoing consumption of C3 and FB, and increased generation of the MAC.

### C3b-FH Complex Modeling

**Figure 2:**
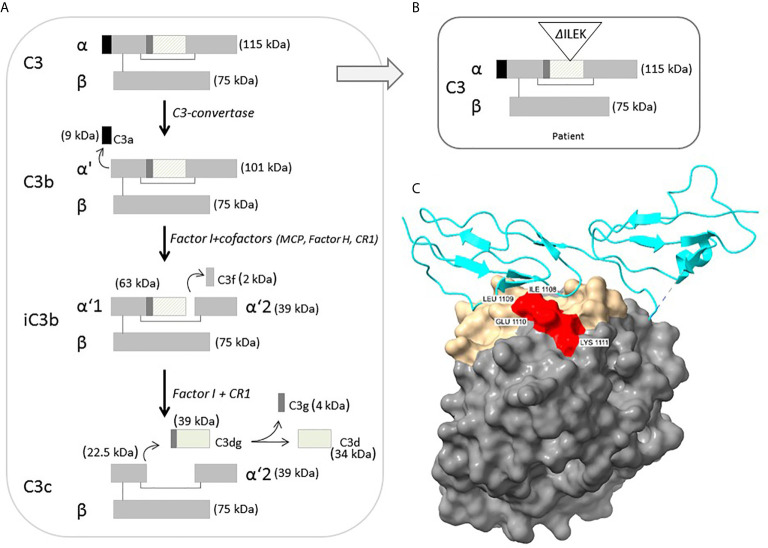
**(A)** Schematic representation of intact C3 structure, processing and regulation. The diagonal-line filled rectangle represents the TED of C3. **(B)** Schematic representation of the position of four-residue deleted sequence (ΔILEK) within the TED domain of the patient’s C3 alpha chain. **(C)** Modelling of the C3b (TED domain)-Factor H complex (from PDB 3OXU) (19), using the UCSF ChimeraX tool. C3b is shown in solvent-excluded molecular surface representation, and Factor H (P08603) in cyan ribbons. All C3b interfacial amino acids are colored gold, while the p.1008-1111 (ILEK) mutant region is colored red. As clearly demonstrated, amino acids ILEK are an integral part of the C3b-Factor H interface.

Structural analysis of C3b-FH complex demonstrates strategic location of the four deleted amino acids (ILEK) within the TED domain, at the C3b-FH interface (**Figure 2C**). The energy difference between wild-type and mutant C3b-FH complex structure (ΔG-wild-type – ΔG mutant) is estimated to be ΔΔG ~ -5Kcal, suggesting a catastrophic effect exerted by the deletion on interface stability. Hence, the ILEK deletion in mutant C3 is highly likely to disrupt C3b-FH interaction, thereby disabling the inhibitory effect of FH on downstream activation of C3b.

## Discussion

We herein described the clinical and molecular investigation in a male infant from a consanguineous family, with neonatal presentation of fulminant aHUS and multi-organ failure, accompanied by extremely low serum levels of C3, a favorable systemic response to eculizumab therapy, and intolerance to eculizumab cessation. Mutation analysis revealed a small homozygous in-frame deletion of 12 bp in exon 26 of the *C3* gene, resulting in four amino acids deletion (ILEK) from the inferred TED of C3, in association with apparent C3 deficiency. However, complement profile analysis indicated unbalanced activation of the ACP, as evidenced by a reduced level of FB denoting its consumption, combined with excessive generation of the terminal MAC. Moreover, structural modelling of C3b-FH complex suggests a disruptive effect of ILEK-deletion on mutant C3b binding to FH, thereby supporting the hypothesis of unbalanced degradation of mutant C3 as underlying the mechanism of aHUS.

C3 is the most abundant complement component protein, playing a crucial role in activation of all three complement pathways. In its mature, unprocessed form, the C3 protein is composed of one α and one β chain linked by a disulfide bond, and consists of 13 domains, harboring intrinsic functional and binding capacity to various effector ligands. Intact C3 is an inert molecule, exhibiting its biological activity only after proteolytic cleavage to functional degradation product (**Figure 2A**). Under physiological condition, C3b is constantly produced at a very low rate, by spontaneous hydrolysis to C3(H2O). The C3b factor is able to attach to pathogens and host cell surfaces, where it binds FB, which in turn is cleaved by complement factor D (FD). The resulting C3bBb or C3 convertase further cleaves and activates C3, thereby leading to an amplification loop of the alternative complement cascade, culminating in MAC-related tissue damage (23, 24).

aHUS secondary to C3 disruption is relatively rare, especially during the pediatric age group, with an overall reported prevalence ranging between 4.5%-11.4 of aHUS cases (3, 4, 25). C3-associated aHUS is mostly attributed to heterozygous gain-of-function variants in the *C3* gene, leading to unbalanced activation of the complement cascade (8, 26–28). Furthermore, the presentation of aHUS in carriers of C3 gain-of-function mutations may be influenced by the presence of the *MCP*ggaac risk haplotype, as previously described (29). In contrast, homozygous loss-of-function variants in C3 are usually implicated in C3 deficiency, manifested by increased susceptibility to recurrent bacterial infections and autoimmunity, unrelated to aHUS (15, 16). To the best of our knowledge, the constellation of aHUS secondary to a small homozygous deletion in *C3* associated with unbalanced activation of the ACP has not been previously reported (OMIM databases).

According to the crystallographic structure of C3, the TED, located within residues 963-1268, contains a hidden thioester bond, which is exposed upon C3 cleavage, thereby facilitating covalent binding of C3b to target surface ligands (30, 31). Moreover, conformational displacement of the TED domain has been previously shown to disrupt the interaction of C3b with the ACP regulators, i.e., FH and MCP (32, 33). Hence, the inferred deletion of four highly conserved residues from the TED of the p.Ile1108_Lys1111del-C3 protein (**Figures 2B, C**), may impose a conformational change of functional significance on the TED, rendering mutant C3b resistant to inactivation, through impaired binding to FH. This hypothesis is further supported by previous reports on the functional significance of various aHUS-related *C3* missense variants within the TED, (i.e., p.L1109V, p.P1114L, p.D1115N, p.G1116R, I1157T), residing in close proximity to the location of p.Ile1108_Lys1111del-C3, with accumulating evidence for their disrupted cell surface interaction with FH, or resistance to inactivation by MCP, due to C3b altered conformation (4, 5, 26, 34, 35).

Of note, hereditary aHUS, in general, is well-known for its partial penetrance, resulting in phenotypic variability among carriers of a shared disease-causing variant. Incomplete penetrance is commonly attributed to the harmful effect of background risk variants within ACP regulatory genes, including *CFH* and *MCP* risk haplotypes (5, 36, 37). The fact that all heterozygous carriers of the p.lle1108_Lys1111del-C3 variant within the studied family are disease-free, suggests that heterozygosity for this likely-pathogenic activating variant, either in the absence or presence of additional risk variants, is insufficient for the development of full-blown disease, albeit intermediated C3 consumption and ACP activation, as evidenced by the complement profile of the patient’s mother.

In conclusion, we have identified a likely-pathogenic homozygous deletion in C3, associated with life-threatening aHUS secondary to over-activation of the ACP. Complement profile analysis suggests a damaging conformational effect of this deletion on the TED of the C3 protein, rendering it susceptible to unbalanced activation due to impaired interaction with regulatory FH. Our findings enable prenatal diagnosis and early treatment initiation in newly identified subjects at risk, while reinforcing the significance of an intact C3-TED for normal function and regulation of the ACP. Yet, the exact mechanism whereby the identified 4-residue deletion in C3 may affect molecular interactions of the C3 protein with additional regulators of the ACP, with ensuing downstream over-activation of terminal complement cascade, warrants further investigation.

## Data Availability Statement

The datasets presented in this study can be found in online repositories. The names of the repository/repositories and accession number(s) can be found below: https://www.ncbi.nlm.nih.gov/genbank/, 2384952.

## Ethics Statement

The studies involving human participants were reviewed and approved by the Rambam Health Care Campus Institutional ethics committee. The patients/participants provided their written informed consent to participate in this study. Written informed consent to participate in this study was provided by the participants’ legal guardian/next of kin.

## Author Contributions

SP: Writing – original draft – Preparation, creation and/or presentation of the published work, specifically writing the initial draft (including substantive translation). DM and ZP: Writing – review & editing – Preparation, creation and/or presentation of the published work by those from the original research group, specifically critical review, commentary or revision – including pre- or post-publication stages. IE, AM, TP, AO, HB-F, KW, NV, RS, FG, and DC: research activity - performed the clinical and laboratory analysis, reviewed and edited the paper. All authors contributed to the article and approved the submitted version.

## Funding

The study was supported by the Premium Postdoctoral Fellowship Program of the Hungarian Academy of Sciences (PPD2018-016/2018), and by the Kaylie Kidney Research Center of Excellence at Rambam Medical Center.

## Conflict of Interest

The authors declare that the research was conducted in the absence of any commercial or financial relationships that could be construed as a potential conflict of interest.
